# Prenatal ultrasonography diagnosis of fetal microtia with tympanic ring agenesis: a case report

**DOI:** 10.3389/fmed.2026.1969602

**Published:** 2026-10-05

**Authors:** Jie Li, Yanpeng Song

**Affiliations:** 1 Department of Imaging, Jiaozhou Central Hospital, Qingdao, Shandong, China; 2 Department of Ultrasound, Qingdao Central Hospital, University of Health and Rehabilitation Sciences (Qingdao Central Hospital), Qingdao, Shandong, China

**Keywords:** absence of the tympanic ring, congenital external and middle ear malformation, CT, fetus, microtia, prenatal ultrasound

## Abstract

Microtia is frequently accompanied by middle ear anomalies, among which absence of the tympanic ring is relatively rare and seldom diagnosed prenatally. Previous reports have mainly focused on external ear anomalies, with limited experience in the prenatal diagnosis and postnatal verification of middle ear osseous abnormalities.We report a case of fetal microtia complicated by absence of the tympanic ring diagnosed by prenatal ultrasound. A systematic examination at 24 weeks of gestation revealed abnormal unilateral auricular morphology, with size and shape inconsistent with a normal fetus at the same gestational age; normal auricular structures were poorly visualized, and the external auditory canal was not clearly demonstrated. On the affected side, the middle ear tympanic ring was not visualized, with absence of its ossification center, suggesting microtia complicated by absence of the tympanic ring. After prenatal counseling, the woman chose to terminate the pregnancy. Post-induction CT confirmed findings consistent with prenatal ultrasound.This case verifies at the histological level that the external and middle ear share a common origin, and demonstrates that prenatal ultrasound can clearly display fetal external and middle ear structures, holding important clinical value for diagnosing microtia with absence of the tympanic ring. Recognition of the ultrasound features of tympanic ring absence may improve prenatal detection and provide a reliable basis for perinatal management and genetic counseling. Prenatal ultrasound is an effective method for diagnosing fetal microtia and assessing concomitant absence of the tympanic ring, helping improve prenatal recognition of occult middle ear anomalies and reduce missed diagnoses. This study clarifies the diagnostic value of postnatal CT verification and provides new practical evidence for the prenatal diagnostic pathway of such congenital anomalies

## Introduction

Fetal microtia combined with tympanic ring agenesis is a clinically rare condition (1, 2). Most existing reports on prenatal diagnosis have focused primarily on the detection of external ear malformations, and experience remains relatively limited regarding the prenatal diagnosis and postnatal confirmation of developmental abnormalities involving the bony structures of the middle ear. Congenital microtia is a morphological abnormality of the external ear caused by abnormal development of the first and second branchial arches during the embryonic period, and it may present as a small, irregularly shaped, or absent auricle (3, 4). Middle ear and external ear are closely related in embryonic development. The tympanic ring, as an important structure for tympanic membrane attachment, its absence often leads to abnormalities of the sound-conducting structures of the middle ear (5). Prenatal ultrasound is the primary means of fetal structural screening. With the application of two-dimensional and three-dimensional ultrasound techniques, the visualization rate of the external ear morphology has gradually improved. However, due to the influence of fetal position, amniotic fluid, and acoustic window conditions, the prenatal visualization of the fine structures of the middle ear remains relatively difficult. There are limited prenatal ultrasound reports in the literature regarding the absence of the fetal tympanic ring. This case demonstrated the simultaneous detection of microtia and tympanic ring agenesis during prenatal ultrasonography,suggesting that upon identifying abnormalities in the external ear morphology, the middle ear structures should be included in the systematic scanning protocol. This report describes the prenatal ultrasound diagnostic procedure, aiming to provide a reference for the prenatal identification of similar cases. We report the prenatal ultrasound diagnostic process in this case, with the aim of providing a reference for the prenatal identification of similar cases. In this case, microtia was diagnosed prenatally by ultrasound, and a combined tympanic ring agenesis was confirmed by CT examination of the specimen after termination of pregnancy. This case clarifies the diagnostic value of post-termination CT verification for middle ear developmental abnormalities detected by prenatal ultrasound, and provides new practical evidence for the prenatal diagnostic pathway of such congenital malformations.

## Case description

A 35-year-old woman, gravida 3,para 2,underwent systematic prenatal ultrasound screening at this hospital at 24 + 3 weeks of gestation. All biometric measurements were consistent with those expected at 24 weeks: biparietal diameter 6.31 cm, femur length 4.52 cm, head circumference 22.45 cm, and abdominal circumference 21.58 cm. The right auricle appeared normal in morphology, measuring approximately 1.80 cm in longitudinal diameter (Figure 1A), whereas the left auricle was abnormal, measuring approximately 0.64 cm (Figure 1B). Transverse section of the skull base demonstrated a well-developed right tympanic ring with visible ossicular chain structures within the tympanic cavity, while the left tympanic ring and ossicular chain were not visualized (Figure 1C). The ultrasound diagnosis was left microtia with ipsilateral tympanic ring agenesis and suspected external auditory canal atresia. The patient subsequently presented to Qingdao Women and Children's Hospital, where ultrasound diagnosed left microtia; however, the development of the tympanic ring was not assessed. The patient had previously been in good health. At 25 + 1 weeks of amenorrhea, she reported having felt fetal movement since the fourth month of pregnancy up to the present. She had no history of infection or fever during pregnancy, no pet exposure, and no vaginal bleeding. She had no history of infectious diseases such as hepatitis or tuberculosis, no history of hypertension, diabetes, trauma, or blood transfusion, no adverse habits such as smoking or alcohol use, and no history of exposure to drugs, toxins, or radiation. Her spouse was in good health and had two sons, both of whom were in good health. Her parents and first-degree relatives were healthy, with no family history of other genetic or infectious diseases. Based on a comprehensive assessment of the patient's condition, the attending physician considered that there were no high-risk factors for TORCH testing. The estimated date of delivery calculated from the early pregnancy ultrasound was accurate. She had attended six prenatal check-ups during pregnancy, and the nuchal translucency (NT) examination at 3 months of gestation was normal. Down syndrome screening indicated an age-related borderline risk, and non-invasive DNA testing indicated low risk. At 24 weeks of gestation, malformation screening ultrasound performed at Qingdao Jiaozhou Central Hospital and Qingdao Women and Children's Hospital revealed left microtia in the fetus. After being informed of the developmental condition, the patient consulted with her family and requested induced labor. On December 21, 2023, a “Rivanol induction of labor” was performed. On December 22, 2023, a female stillborn infant was delivered vaginally, weighing 750 g. The placenta and membranes were incompletely delivered, and curettage was performed. Postpartum anti-inflammatory treatment consisted of cefazolin 1.0 g by intravenous drip twice daily for 2 days, and oral Yimucao capsules 3 capsules three times daily as a uterine contraction-promoting agent. Follow-up postpartum ultrasound showed no abnormalities.

**Figure 1:**
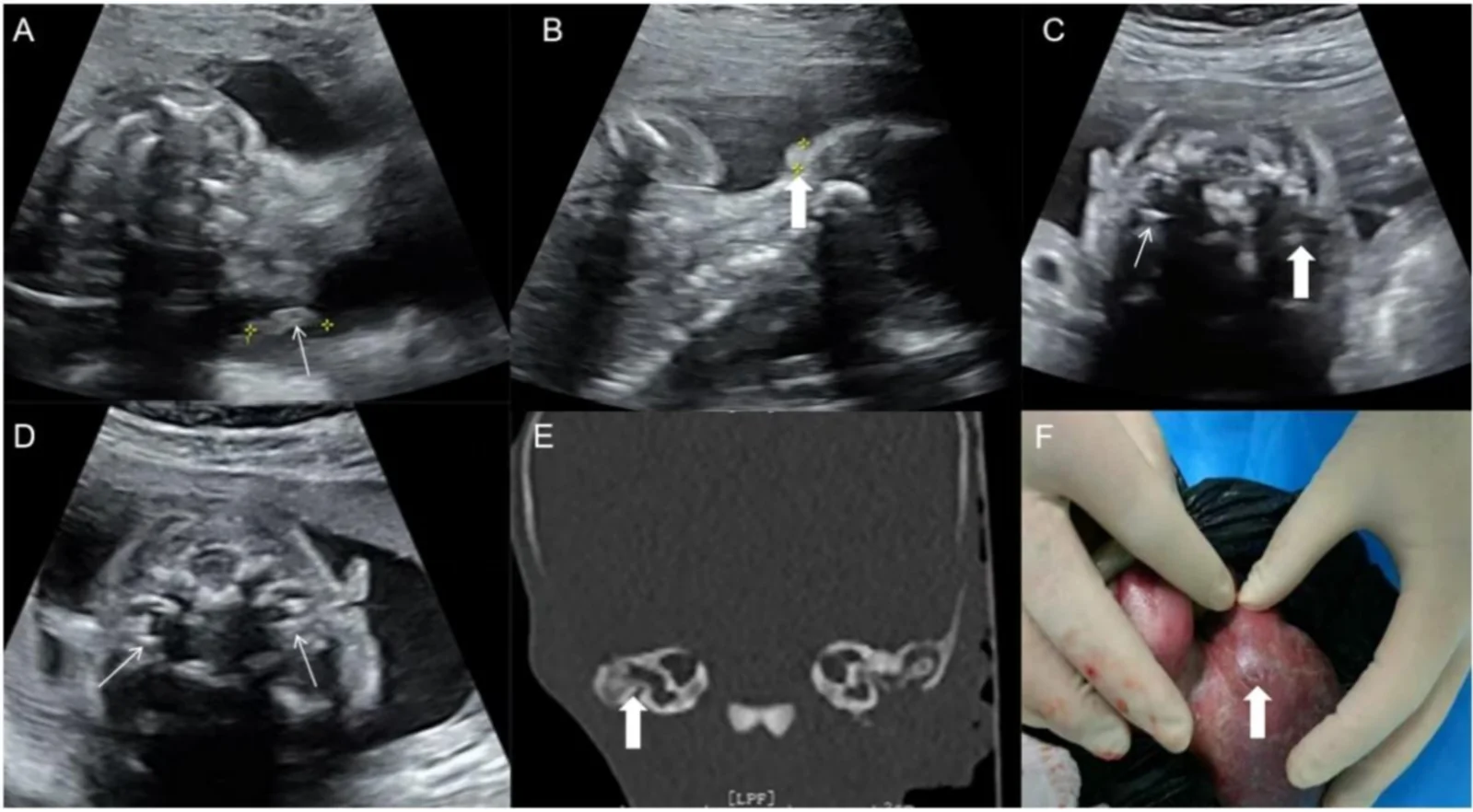
**(A)** normal right auricular region. **(B)** Abnormal left external ear (microtia). **(C)** Craniocaudal cross-sectional view: the left typanic ring is absent, whereas the right tympanic ring is normal. **(D)** Standard skull base cross-section: the left tympanic ring is symmetrical to the right. **(E)** Coronal CT scan reveals absence of the tympanic ring and ossicular chain; the right tympanic ring and ossicular chain appear normally developed. **(F)** Induced labor fo a fetus with left-sided microtia. (Fine arrows indicate normal structures; thick arrows indicate abnormal structures.).

Gross examination of the specimen after induced labor showed that only a remnant of the external ear was present on the left side of the stillborn fetus, with absence of auricular tissue; a probe could not be passed into the external auditory canal, consistent with microtia with external auditory canal atresia (Figure 1E). After obtaining consent and authorization from the pregnant woman and her family, a cranial CT scan of the induced labor specimen was performed. Coronal views showed absence of the left tympanic ring with absence of the ossicular chain and non-visualization of the external auditory canal, while the right tympanic ring and ossicular chain appeared normal; the bilateral bony labyrinth was well developed, consistent with left tympanic cavity absence with external auditory canal atresia (Figure 1F). This study was reviewed and approved by the hospital ethics committee (QSSJZZXYY Approval No. 202312-01) (Figure 2).

**Figure 2:**
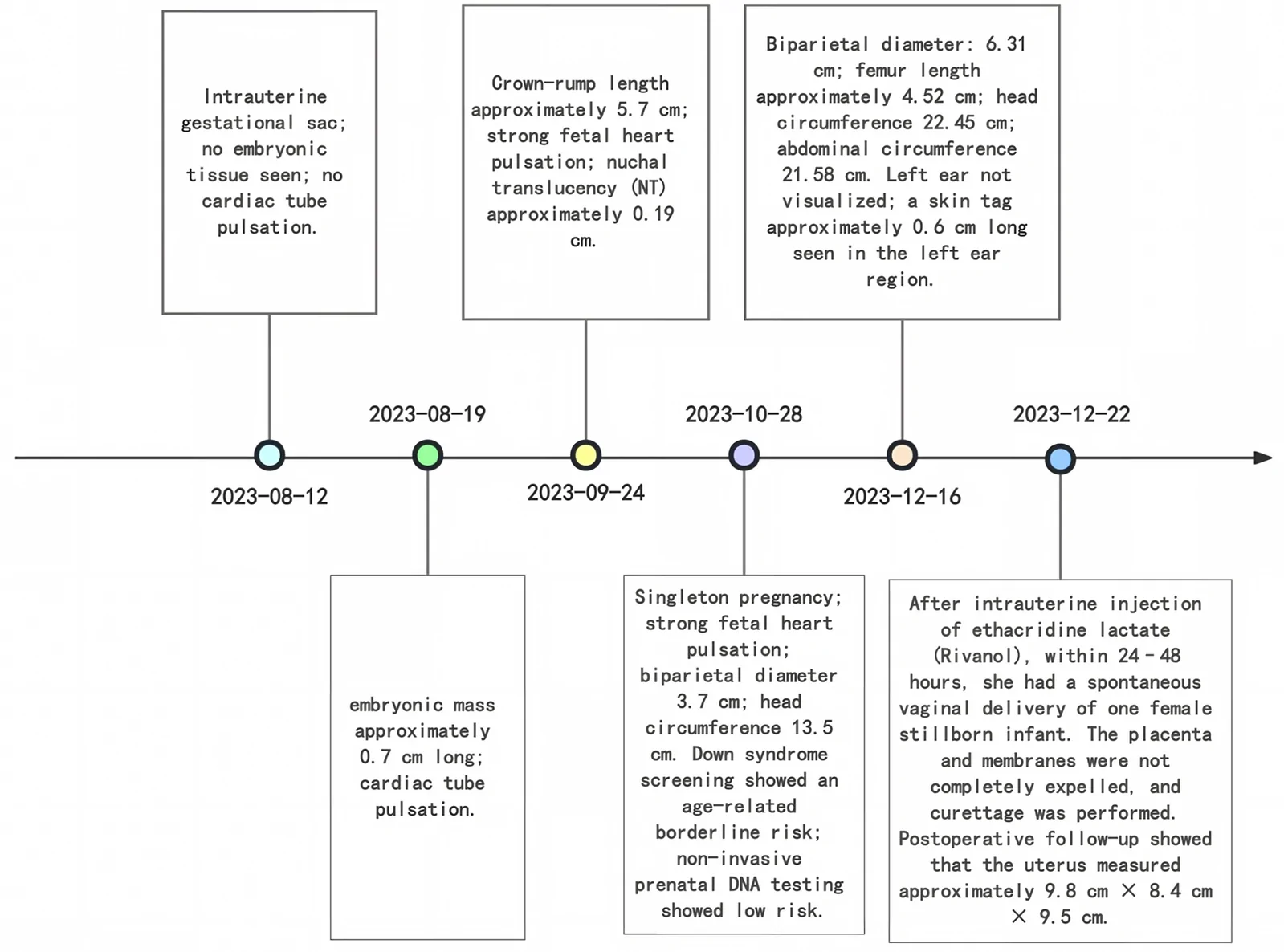
Timeline of patient visit and treatment-related data.

## Discuss

During embryonic development, the middle ear and external ear originate from the same embryonic tissues (6), both arising from the first and second branchial arches, which are formed by the migration of neural crest cells from the posterior midbrain and rhombomeres R1-R5. The fetal auricle is formed by several prominences of the first and second branchial arches surrounding the first branchial cleft, while both the external auditory canal and the tympanic ring originate from the first branchial cleft between the first and second branchial arches; the tympanic ring develops from the mesenchyme of the first branchial arch (6, 7). M. Moll demonstrated that the development of the external auditory canal depends on the tympanic ring, and that when the tympanic ring is absent, the external auditory canal is also absent (8). As part of the middle ear, the tympanic ring is associated with malformations of the auricle and external auditory canal when its development is abnormal (2). Clinically, external and middle ear malformations also frequently occur simultaneously, presenting as microtia, external auditory canal stenosis or atresia, and middle ear malformation, with conductive hearing loss as the auditory manifestation (9). Microtia is one of the most common facial malformations, second only to cleft lip and palate, and its incidence on the right side (approximately 53%-66%) is significantly higher than on the left; it is often accompanied by external auditory canal atresia and middle ear malformation (10). Microtia is classified into 4 grades (11): Grade 1: the auricle is slightly small but its contour is basically normal; Grade 2: the auricle is present but slightly small, with abnormal anatomical structures; Grade 3: there is no auricular morphology, only a cord-like soft tissue structure, with no external auditory canal, often accompanied by middle ear malformation; Grade 4: there is almost no trace of the auricle and no external auditory canal. In this fetus, the left auricle appeared only as a fleshy protuberance on the long-axis view, and was nearly absent on the transverse section of the skull base (Figure 1C), placing it within the Grade 3 category of the above malformation classification.

Leibovitz et al. (1) were the first to report that prenatal ultrasound can evaluate the structure of the fetal tympanic ring. Based on the standard transverse section of the skull base described in the literature, the author demonstrates the following: the tympanic ring appears as a mirror-symmetric, “C"-shaped, thin-walled bony hyperechoic structure, located on either side of the basilar part of the occipital bone; within the tympanic ring, a linear bony hyperechoic structure can be clearly visualized, namely the ossicular chain (Figure 1D); on this section, the anteroposterior and transverse diameters of the bilateral tympanic rings can be measured, and the development of the ossicular chain can also be assessed. In this fetus, the transverse section of the skull base showed absence of the anatomical structures of the left tympanic ring and ossicular chain, while the anatomical structures of the right tympanic ring and ossicular chain were well visualized. Cranial coronal CT of the post-induced-labor specimen further confirmed the accuracy of prenatal ultrasound in evaluating the tympanic ring. External and middle ear malformations are among the common manifestations of chromosomal abnormalities (12). Nuñez-Castruita A proposed that the tympanic ring may be a reliable and sensitive indicator of abnormal development of the external and middle ear; they used autopsy to demonstrate the morphology of the fetal tympanic ring and its developmental pattern (13), but did not use ultrasound and clinical cases to confirm the relationship between the tympanic ring and external auditory canal abnormalities and microtia. In this case, ultrasound examination was used to diagnose microtia with tympanic ring agenesis, and CT examination after induced labor confirmed this diagnosis, suggesting that there may be an association between microtia and tympanic ring agenesis.

Previous prenatal ultrasound reports on microtia have mostly focused on the morphology of the auricle and the measurement of its dimensions (13), with few studies reporting on the external auditory canal and middle ear structures (tympanic ring). Some scholars have found that the fetal tympanic ring is a reliable indicator of external and middle ear developmental abnormalities. The anteroposterior diameter of the tympanic ring increases with advancing gestational age and with increasing auricular length. Ultrasound assessment of the tympanic ring is optimal during the second trimester. Evaluation of tympanic ring development can help clinicians address the challenge of prenatal diagnosis of external auditory canal atresia and stenosis, and facilitates multidisciplinary prenatal and postnatal counseling (2). In this case, prenatal ultrasound first identified microtia as the clue, and further scanning simultaneously detected absence of the ipsilateral tympanic ring and ossicular chain on the transverse section of the skull base, which is consistent with the findings reported in the literature (13), further confirming that tympanic ring agenesis coexists with external auditory canal atresia, indicating a correlation between the two.

Prenatal ultrasound has irreplaceable value in the diagnosis of fetal microtia with tympanic ring agenesis (14, 15). Its value is mainly reflected in the following aspects: First, it enables early detection of the malformation, providing key information for prenatal genetic counseling and pregnancy decision-making. Second, it allows preliminary grading of the severity of the malformation by assessing the residual auricular structures, the patency of the external auditory canal, and the integrity of the tympanic ring, thereby providing a prediction for postnatal treatment planning. Third, it enables comprehensive assessment of whether other systemic malformations are present, helping to determine whether the microtia is syndromic, which directly affects prognostic evaluation. However, prenatal ultrasound also has obvious limitations. Due to factors such as the small size of the tympanic ring, fetal position, oligohydramnios, obstruction by fetal limbs, and increased acoustic shadowing of the skull in late pregnancy, the visualization rate is not 100%. In addition, diagnostic accuracy is highly dependent on the operator's experience, equipment resolution, and whether standardized scanning planes are used, and false-negative and false-positive cases should not be overlooked. Partial tympanic ring agenesis is particularly prone to being missed, as its subtle interruption of bony structures is difficult to distinguish from acoustic beam artifacts on two-dimensional ultrasound.

When prenatal ultrasound suggests an absent tympanic ring, it must be distinguished from normal ossification variants, temporal bone dysplasia, and artifact-related pseudodisruption. Given current evidence and clinical needs, the tympanic ring deserves greater attention in prenatal practice. We recommend including its scanning in routine mid-trimester systematic ultrasound screening, with dedicated targeted scanning whenever auricular or external auditory canal abnormalities are seen. Standardized scanning protocols and reporting templates should also be developed to refine its description. When an absent tympanic ring is detected, a multidisciplinary pathway involving otology, pediatric maxillofacial surgery, and genetic counseling should be established to ensure continuity from prenatal diagnosis to postnatal evaluation and intervention (16–18). Future large-sample, multicenter prospective studies are needed to assess the sensitivity, specificity, and predictive value of prenatal ultrasound for this condition, and to correlate prenatal findings with postnatal hearing outcomes, surgical management, and long-term quality of life, thereby enhancing the clinical utility of prenatal imaging.

## Data Availability

The original contributions presented in the study are included in the article/Supplementary Material, further inquiries can be directed to the corresponding author.
